# The Journey to Diagnose Spondyloarthritis in Patients From Riyadh: A Cross-Sectional Study

**DOI:** 10.7759/cureus.29951

**Published:** 2022-10-05

**Authors:** Mohamed K Bedaiwi, Mohammad T Nouri, Majed A AlJohani, Abdulaziz A Aljohani, Abdullah H AlOmar, Sultan N Alnasser, Mohammed O Alomar

**Affiliations:** 1 Rheumatology, Internal Medicine, King Khalid University Hospital, King Saud University, Riyadh, SAU; 2 College of Medicine, King Saud University, Riyadh, SAU; 3 Medicine, King Saud University, Riyadh, SAU; 4 Orthopedics, King Khalid University Hospital, Riyadh, SAU; 5 Dermatology, King Khalid University Hospital, Riyadh, SAU

**Keywords:** riyadh, cross sectional study, diagnosis, delay, spondylarthritis, ankylosing spondylitis

## Abstract

Introduction

Ankylosing spondylitis, now frequently referred to as spondyloarthritis (SpA), is a chronic inflammatory disease causing axial arthritis and inflammatory lower back pain resulting in the eventual impairment of spinal mobility. Moreover, its systemic complications include stiffness and inflexibility, restriction of lung capacity and function, eye inflammation, compression spinal fractures, and heart problems. Hence, early diagnosis and intervention play a key role in preventing acute complications and improving the quality of life.

Objective

We aimed to estimate the average duration of diagnosis, the average number of doctors visited, and the association between the specialty of the first physician and the length of SpA diagnosis delay.

Methods

A cross-sectional retrospective study was conducted from November 2019 to April 2020 with patients from King Khalid University Hospital, Riyadh, Saudi Arabia. The patients were 18 years and older and diagnosed with SpA. Call interviews were conducted and patients’ medical charts were reviewed. The data were analyzed using the Statistical Package for Social Sciences statistical software, version 23 (IBM Corp., Armonk, NY).

Result

The total sample was 101 patients: 59 (58.4%) males and 42 (41.6%) females. The average duration from the onset of symptoms until seeking medical advice (lag 1) and from seeking medical advice until the definite diagnosis (lag 2) was 24.74 ± 48.13 and 16.16 ± 34.62 months, respectively. The average number of doctors visited between the first medical encounter and the final diagnosis was 3.56 ± 5.3. Patients who consulted rheumatologists as the first medical encounter showed less delay in diagnosis compared to patients who sought non-rheumatologists, such as orthopedists, emergency physicians, and general physicians (11.81 ± 33.35 months vs. 26.63 ± 44.28, 26.96 ± 44.88, and 44.33 ± 65.75 months, respectively).

Conclusion

Patients with SpA who were not seen by rheumatologists took a longer period till the final diagnosis than those who visited rheumatologists earlier in the course of the disease. Therefore, more studies are required to define the exact factors leading to the delay.

## Introduction

Ankylosing spondylitis (AS), now frequently referred to as spondyloarthritis (SpA), is a chronic inflammatory disease causing axial arthritis. The disease commonly results in inflammatory low back pain, with eventual impairment of spinal mobility due to structural changes, spinal fusion, and peripheral arthritis [1]. The prevalence of AS varies from 9-30 per 10,000 in the population, depending on the geographic area [2-8]. Axial SpA epidemiology data in the Middle East are scarce although one study indicated a psoriatic arthritis prevalence of 0.01% [9].

Regarding the risk factors of AS, there is no known specific cause although people with the HLA-B27 gene are at an increased risk of developing AS. Moreover, the male gender, late adolescence, and early adulthood are factors that appear to increase the risk. In acute SpA, there are many complications such as stiffness and inflexibility, lung capacity and function restriction, eye inflammation, compression spinal fractures, and heart problems [10].

Early diagnosis and intervention play a key role in preventing acute complications and improving the quality of life [11-13]. Early diagnosis can be achieved by earlier recognition of symptoms associated with AS features such as uveitis, enthesitis, HLA-B27 expression, and disease severity metrics [14-16]. Unfortunately, patients with AS are ranked the highest in late diagnosis compared to patients with other rheumatic diseases [16].

In Saudi Arabia, the exact factors associated with a delay in diagnosis of patients with SpA remain unknown. Defining the possible factors leading to the late diagnosis and treatment of patients with SpA in Saudi Arabia may ultimately improve disease outcomes. Thus, a detailed understanding of patients with SpA is required. Therefore, this study aims to estimate the duration of diagnosis in patients with SpA and the possible factors leading to a delayed diagnosis.

## Materials and methods

Study design and setting

A cross-sectional retrospective study was conducted between November 2019 and April 2020 with 101 patients from King Khalid University Hospital, Riyadh, Saudi Arabia, using simple random sampling for a single mean study. The research was approved by the Health Sciences Colleges Research on Human Subjects King Saud University College of Medicine, project number E-19-4426.

Data collection

The patients’ medical charts were reviewed, and four research assistants conducted call interviews. The questionnaire was directly adopted from previous data published by Waleed Hussain in 2015 [17]. To avoid bias, the interviewer acquired standardized training for interview questions. The informed consent information was explicit and confirmed the study’s intent, stating the participants’ right to withdraw from the study at any time without obligation. Participants’ anonymity was assured by assigning each participant a code number for analysis.

Questionnaire for the charts review and call interviews

The following demographics were evaluated utilizing the medical charts and call interviews: gender, age, educational level, the duration of time from the onset of symptoms to the first consultation, the duration of time from the initial consultation to the definite diagnosis, the number of doctors visited by patients with SpA before the definite diagnosis was made, the specialty of the first doctor visited, and the specialty of the doctor who made the definite diagnosis. The patient’s first medical encounter with SpA or its related symptoms was determined during the phone interview, and the initial definitive SpA diagnosis was defined as the first time the responsible doctor told each patient they had SpA. The dates that patients identified during the phone interview were matched with the dates of their final diagnosis in their medical history. If the specific time of the first medical visit or symptom was unknown, the patient estimated the interval. In addition, any symptoms or clinical features not mentioned by the patient during the phone interview or absent in the medical history were not considered.

Participants in the interview/chart review

The patients were 18 years and older and diagnosed with SpA. Patients under 18 or who refused to participate were excluded from the study. The study followed ethical standards, and oral informed consent was obtained from each patient.

Statistical analysis

All data were analyzed using the Statistical Package for Social Sciences software, version 23 (IBM Corp., Armonk, NY). All categorical variables were presented as numbers and percentages, including the frequencies of the nominal variables, the mean, and the standard deviation. The quantitative variables included age, BMI, the duration from the onset of symptoms to the first consultation (lag 1), the duration from the initial consultation to the definite diagnosis (lag 2), the delay in diagnosis (from the onset of symptoms to the definite diagnosis (total lag)), and the number of doctors. A t-test was used to compare lag 2 and the total lag with gender, fatigue, the number of visited doctors, and the level of education (before college = low education, college and above = high education). Analysis of variance (ANOVA) was used to compare more than two groups (e.g., various specialty doctors and the average for all specialty doctors).

## Results

A total of 101 patients were diagnosed with SpA, and all participated in the call interviews (response rate = 100%). Among them, 59 (58.4%) were males, 42 (41.6) were female, and 96 (95.0%) were Saudi while five (5.0%) were non-Saudi (mean age 39.86 ± 12.08 years). Participants’ average BMI was 23 ± 5 (29.01 ± 6.53). Moreover, 76 (75.2%) were married, 23 were (22.8%) single, and two (2.0%) were divorced. Furthermore, 66 (65.3%) had high education while 35 (34.7%) had low education.

More than half the patients, 70 (69.3%), showed symptoms of fatigue. Only 37 (36.6%) had their first visit with a rheumatologist while 17 (16.8%), 14 (13.9%), six (5.9%), eight (7.9%), eight (7.9%), three (3.0%), and eight (7.9%) had the first visit with non-rheumatologists-orthopedists, emergency physicians, dermatologists, family medicine physicians, internal medicine, general physicians, and others, respectively. The internal diagnosis of most patients (85 [84.1%]) was AS while others were diagnosed with psoriatic arthritis (14 (13.9%)), one (1%) was diagnosed with Crohn’s disease, and one (1.0%) was diagnosed with ulcerative colitis. Most (88 (87.1%)) had a definite diagnosis by a rheumatologist while 13 (12.9%) were diagnosed by non-rheumatologists (Table 1).

**Table 1:** Sample characteristics n: frequency, %: percentage, x: mean, SD = standard deviation

	N (%) 101 (100.0)
Gender:	
Females	42 (41.6)
Males	59 (58.4)
Nationality:	
Saudi	96 (95.0)
Non-Saudi	5 (5.0)
Age (x ± SD)	39.86 ± 12.08
BMI (x ± SD)	29.01 ± 6.53
Marital status:	
Married	76 (75.2)
Single	23 (22.8)
Divorced	2 (2.0)
Education:	
High education	66 (65.3)
Low education	35 (34.7)
Presence of fatigue:	
Yes	70 (69.3)
No	31 (30.7)
The patient’s first specialty doctor:	
Rheumatologist	37 (36.6)
Orthopedist	17 (16.8)
ER	14 (13.9)
Dermatologist	6 (5.9)
Family medicine	8 (7.9)
Internal medicine	8 (7.9)
General physicians	3 (3.0)
Others	8 (7.9)
Internal diagnosis:	
Ankylosing spondylitis	85 (84.1)
Psoriatic arthritis	14 (13.9)
Crohn's disease	1 (1.0)
Ulcerative colitis	1 (1.0)
The specialty of the doctor who made the definite diagnosis:	
Rheumatologist	88 (87.1)
Non-rheumatologists	13 (12.9)

The average duration from the onset of symptoms until seeking medical advice (lag 1) was 24.74 ± 48.13 months while the average duration from seeking medical advice until the definite diagnosis (lag 2) was 16.16 ± 34.62 months. The average number of doctors visited between the first medical encounter and the final diagnosis was 3.56 ± 5.3 (Table 2).

**Table 2:** Disease duration: lag 1, lag 2, and number of doctors n: frequency, %: percentage, x: mean, SD = standard deviation

N (%) 101 (100.0)	x ± SD
Disease duration: (Lag 1) (months)	24.74 ± 48.13
Disease duration: (Lag 1) (years)	2.06 ± 4.01
Lag time between first medical encounter until definite diagnosis (Lag 2) (months)	16.16 ± 34.62
Number of doctors visited between the first medical encounter until the final diagnosis was made	3.55 ± 5.32

Patients who consulted rheumatologists as the first medical encounter showed less delay in diagnosis compared to patients who sought non-rheumatologists such as orthopedists, emergency physicians, and general physicians (11.81 ± 33.35 months vs. 26.63 ± 44.28, 26.96 ± 44.88, and 44.33 ± 65.75 months, respectively). In comparison, patients who consulted family medicine physicians, internal medicine physicians, and dermatologists showed the lowest durations of SpA diagnosis delay (6.00 ± 5.52, 9.27 ± 16.18, and 16.33 ± 20.38 months, respectively; Table 3). However, regarding the previous result, only a few patients consulted physicians from these specialties. Hence, these findings were not significant.

**Table 3:** Lag between the first medical encounter until the definite diagnosis (lag 2 in months) n: frequency, %: percentage, x: mean, SD = standard deviation

N (%) 101 (100.0)	N	Percentages	x ± SD
Rheumatologist	37	36.6	11.81 ± 33.35
Orthopedist	17	16.8	26.63 ± 44.28
ER	14	13.9	26.96 ± 44.88
Dermatologist	6	5.9	16.33 ± 20.38
Family Medicine	8	7.9	6.00 ± 5.52
Internal medicine	8	7.9	9.27 ± 16.18
General physician	3	3.0	44.33 ± 65.75
Others	8	7.9	1.45 ± 1.42
Total	101	100	16.16± 34.62

## Discussion

According to our study, the average duration of a SpA diagnosis in Riyadh is 3.4 years. Interestingly, this value is much lower than in Japan, India, and Turkey, with an average of 6.7, 6.9, and 6.05 years, respectively [16,18,19]. Moreover, a study in France found a mean diagnostic delay of 4.9 years [20], lower than many other countries but still much higher than our findings. The exact reason for faster diagnosis in Riyadh has not yet been identified; hence, more studies are required. We divided the length of diagnosis into two parts: the duration from the onset of symptoms until seeking medical advice (lag 1) and the time before the definite diagnosis was made (lag 2).

In our study, lag 1 was 2.06 ± 4.01 years and lag 2 was 1.3 ± 2.8 years. Thus, we concluded from these results that the most significant reason for the delay was patients waiting before seeking medical advice, explained by the insidious nature of the disease and the fact that the extensive use of NSAIDs masks SpA symptoms [21]. Hence, education can play a significant role in decreasing the duration of diagnosis. Nevertheless, physicians still have a considerable role in the delay of diagnosis, as demonstrated by a lack of familiarity with symptoms of back pain specific to SpA [22]. In addition, there is low awareness of the disease by medical professionals compared to more common back disorders [16]. On average, our patients consulted four doctors to reach a final diagnosis. A rheumatologist made a definite diagnosis in 88 of 101 patients. However, only 37 of these patients visited a rheumatologist as the first physician while the other 51 were referred.

Patients who visited a rheumatologist as the first physician had a shorter lag 2 (the duration from seeking medical advice until the definite diagnosis), an average time of 11.8 months, compared to patients who visited non-rheumatologists as the first physician, with an average period of 18.7 months. Notably, laboratory availability and serum utility also play roles in the duration of diagnosis, and imaging biomarkers are limited in SpA [23]. However, currently, MRI is thought to be the most sensitive technique to diagnose SpA [24]. Therefore, after using MRI, which approximately began in 2000, patients with SpA experiencing disease onset after 2000 had a shorter delay diagnosis than those before 2000 [18].

Our study has some limitations, including its retrospective observation. Bias could also have resulted from patients forgetting the exact time of the onset of symptoms, influencing our outcomes. However, we attempted to minimize recall bias using trained interviewers during the patient interviews and sought to identify the exact date of the onset of patient symptoms. Moreover, we matched patient information from the interviews with hospital medical records. Notably, not all factors influencing the diagnosis delay in patients with SpA were included in the analysis.

## Conclusions

Our findings indicate a shorter duration of diagnosis than found in the literature. However, we still found a delay in diagnosis, both in Riyadh and other regions, possibly due to patient delay in seeking medical advice and physician delay in reaching a definite diagnosis. However, rheumatologists reached definite diagnoses faster than non-rheumatologists. Hence, patients with SpA who did not first consult a rheumatologist or were not referred by a non-rheumatologist quickly enough resulted in a longer duration of diagnosis. Therefore, more studies are required to define the exact factors leading to delays. Nevertheless, it appears that early consultation or referral to a rheumatologist accelerates the diagnosis of SpA.

## Appendices

**Table 4:** Questionnaire

1	Demographic Data Domain		
Region:			
	Age: years old.	Nationality:	
	Gender: Male Female		
	Educational level Illiterate Read and write Primary school intermediate school High School college/above		
	Marital status: marital status marital status marital status Married single Divorced widow		
2	Clinical data domain		
	Main Diagnosis:		Disease duration: *time between first symptom and first medical encounter (lag 1) *
	What is the first symptom		
	presence of fatigue? Yes No		
	The patient’s first visit to a specialty doctor		
	The specialty of the doctor who made the definite diagnosis.		
	Lag time between first medical encounter until definite diagnosis (lag 2)		
	Number of doctors visited between the first medical encounter until the final diagnosis was made:		
